# Application of Bronchoscopic TransParenchymal Nodule Access in tuberculous bronchial occlusion

**DOI:** 10.1111/crj.13544

**Published:** 2022-10-24

**Authors:** Qiusheng Jing, Zhimin Hu, Mingdi Wu, Min Liu, Panli Yu, Ling Sun, Xianxiang Chen

**Affiliations:** ^1^ Wuhan Institute for Tuberculosis Control and Cure Wuhan Pulmonary Hospital Wuhan China

**Keywords:** bronchus, BTPNA, occlusion, tuberculosis

## Abstract

Bronchoscopic TransParenchymal Nodule Access (BTPNA) technology is mainly used for sampling or ablative treatment of lung parenchymal lesions that cannot be reached by bronchoscopy and its appendages, generally for palliative treatment of some lung tumors. We used BTPNA to treat a 32‐year‐old young woman with pulmonary tuberculosis and successfully perforated her occluded left main bronchus. Her left atelectasis was recovered, and a silicone stent was inserted to preserve the shape of the left main bronchus.

## Graphical abstract

BTPNA can be used to obtain lung nodules, and interventional vapour or radiofrequency ablation can be used to treat solitary lung tumor. Tunnelling technology can also be used to treat bronchial occlusion and open the airway.

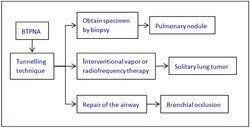

## 1 INTRODUCTION

Tuberculosis is a global public health problem; although the world has made progress in controlling it,^1^ some forms of tuberculosis, such as tracheobronchial tuberculosis, are still very troublesome.^2^ Bronchial tuberculosis has a long incubation period, long course of treatment, and many complications. Among them, bronchial stenosis is the most common.^3^ Bronchial occlusion is a rare complication of bronchial tuberculosis.^4^ If bronchial stenosis is left untreated, atelectasis may occur in the lung to which it belongs^5^, ^6^; the patient's lung function will gradually decline,^2^ and the treatment of tuberculosis will become more and more difficult.^7^ As a result of the balance of the thorax, the thorax will collapse,^8^ the left and right sides of the thorax are asymmetric, and then, the load of the heart will increase and limit the patient's ability to move, reducing the quality of life. Bronchoscopic intervention is the best treatment for bronchial lumen and structural changes caused by bronchial tuberculosis.^9^ For tuberculous bronchial occlusion,^10^ we adopted Bronchoscopic TransParenchymal Nodule Access (BTPNA) technology,^11^, ^12^, ^13^ successfully opened up a case of left main bronchial occlusion, and implanted a stent. The patient's left atelectasis was recovered.

## 2 BTPNA METHODOLOGY

BTPNA was first described in the paper published on CHEST in 2014 by Silvestri et al.^12^ Based on CT scan, LungPoint virtual bronchoscopy navigation software can virtual the route and puncture point of bronchoscopy and accessories in the bronchus. The joint location of magnetic navigation system + C‐arm X‐ray + radial ultrasound, accurate guidance can safely avoid blood vessels. A tunnel is created in the wall of the lumen by special needling and small balloon dilatation, through which various biopsy instruments or probes are sent for sampling or ablation of lung nodules. At present, this technique is mainly used in the diagnosis and palliative treatment of some lung nodules. These lesions are not reachable by conventional transbronchial lung biopsy and belong to non‐natural channel lesions. The treatment described in this paper mainly uses the tunneling function of this technology.

## 3 CLINICAL TIME COURSE

A 32‐year‐old female patient: She was admitted to the hospital on September 23, 2021, with intermittent cough and expectoration for more than 3 months.

**FIGURE 1:**
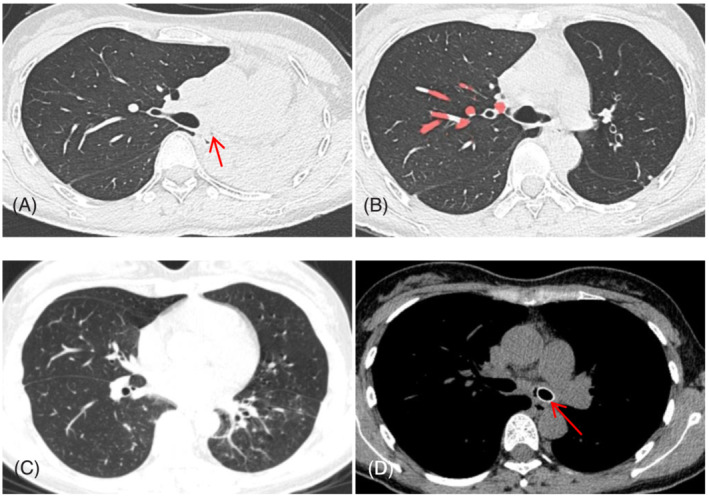
(A) Sep.23.2021, left main bronchial occlusion with left atelectasis (arrow: disappearing left main bronchial remnant); (B, C) Oct.09.2021, the left main bronchial lumen was revealed and the left atelectasis temporarily recovered. (D) Mar.16.2022, the left main bronchus was stented, the lumen was clear, and the left atelectasis recovered (arrow: section image of silicone stent).

**FIGURE 2:**
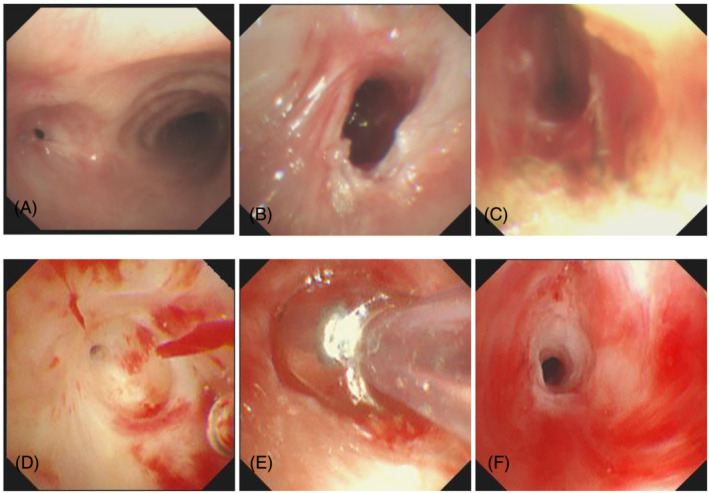
Sep.24.2021, (A) occlusion of the left main bronchus; (B) the left main bronchial opening was significantly enlarged after laser and cryotherapy; (C) lumen of the upper segment of the left main bronchus increased after balloon dilation; Oct.09.2021, (D) the distal end of the left main bronchus was occluded; (E) small balloon dilation; (F) the lumen at the end of the left main bronchus is exposed after BTPNA.

Previous medical history: Anti‐tuberculosis treatment (2HRZE/16HRE) was given after the diagnosis of tuberculosis and tuberculous pleurisy in January 2019, and the drug was stopped 18 months later on the doctor's advice. The lab reported a normal blood routine and coagulation. Primary outpatient bronchoscopy revealed severe stenosis of the left main bronchus (Figure 2A). Clinical diagnosis are as follows: secondary pulmonary tuberculosis, left upper and lower lung and right lower lung [smear (−) culture (−)] stable, left main bronchial tuberculosis with severe lumen stenosis, left atelectasis (Figure 1A). After a comprehensive consultation between clinicians and endoscopicians, it was decided to use bronchoscopy combined with multiple airway interventional techniques to expand the left main bronchus and recover the left atelectasis as much as possible. We will use the latest BTPNA technology for bronchoscopic intervention. The technology has been reviewed and approved by the hospital's medical ethics committee. The intention and purpose of BTPNA therapy were explained to the patient and family, and informed consent was signed by the patient and family.

Before the bronchoscopic intervention, we prepared some relevant medical instruments: (1) one Olympus 1TQ290 and one Olympus MP290F electronic bronchoscope; (2) one FlexNeedle puncture needle and one expanded balloon catheter with a diameter of 4 mm × 6 mm (manufactured by Hangzhou Broncus Medical Co., Ltd.); (3) one expansion balloon catheter with a diameter of 8 mm × 40 mm and one covered stent with a diameter of 10 mm × 40 mm; one silicone stent with a diameter of 10 mm × 40 mm (manufactured by Micro‐tech [Nanjing] Co., Ltd.); (4) Lungpro augmented reality Optical Whole Lung Diagnostic and Clinical Guidance system (Archimedes, Manufactured by Hangzhou Broncus Medical Co., Ltd.); (5) drugs: 2% lidocaine, hemocoagulase; and (6) a YAG laser therapeutic apparatus (model: Ligensisi‐MY100C, Wuhan Ligendsis Technology Co., Ltd. production) and a cryotherapeutic machine (Germany Aierbo company production).

**FIGURE 3:**
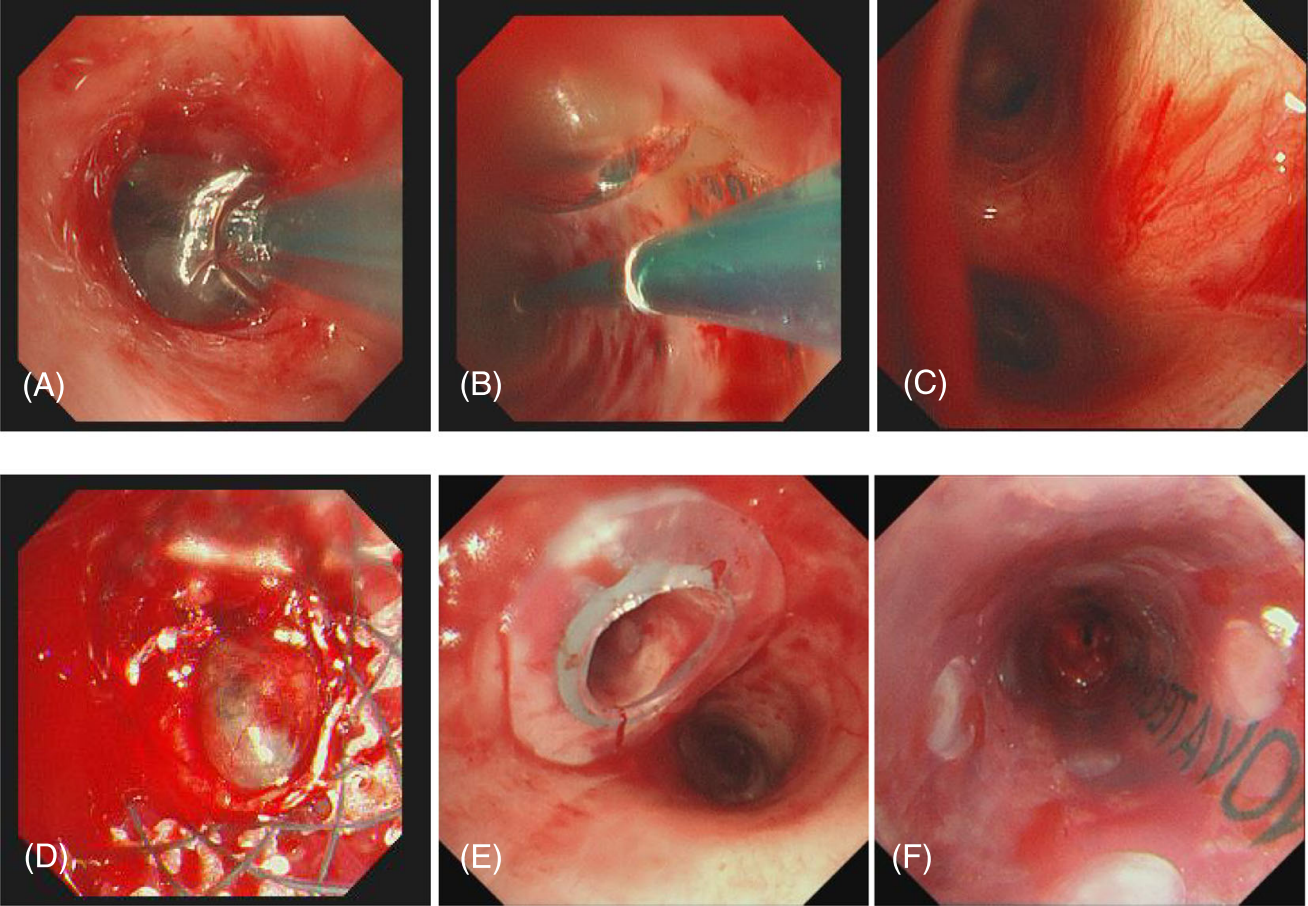
Oct.26.2021, (A) balloon dilation in progress; (B) the internal field of vision of balloon can dynamically observe the state of bronchial wall tearing and bleeding; (C) the opening of the left upper and lower bronchus; (D) the lumen of the left bronchial terminus is unclear after covered stent placement. Nov.20.2021, (E) labial cover with silicone stent at the opening of the left main bronchus; (F) the lumen of left main bronchus is shaped by silicone stent.

**FIGURE 4:**
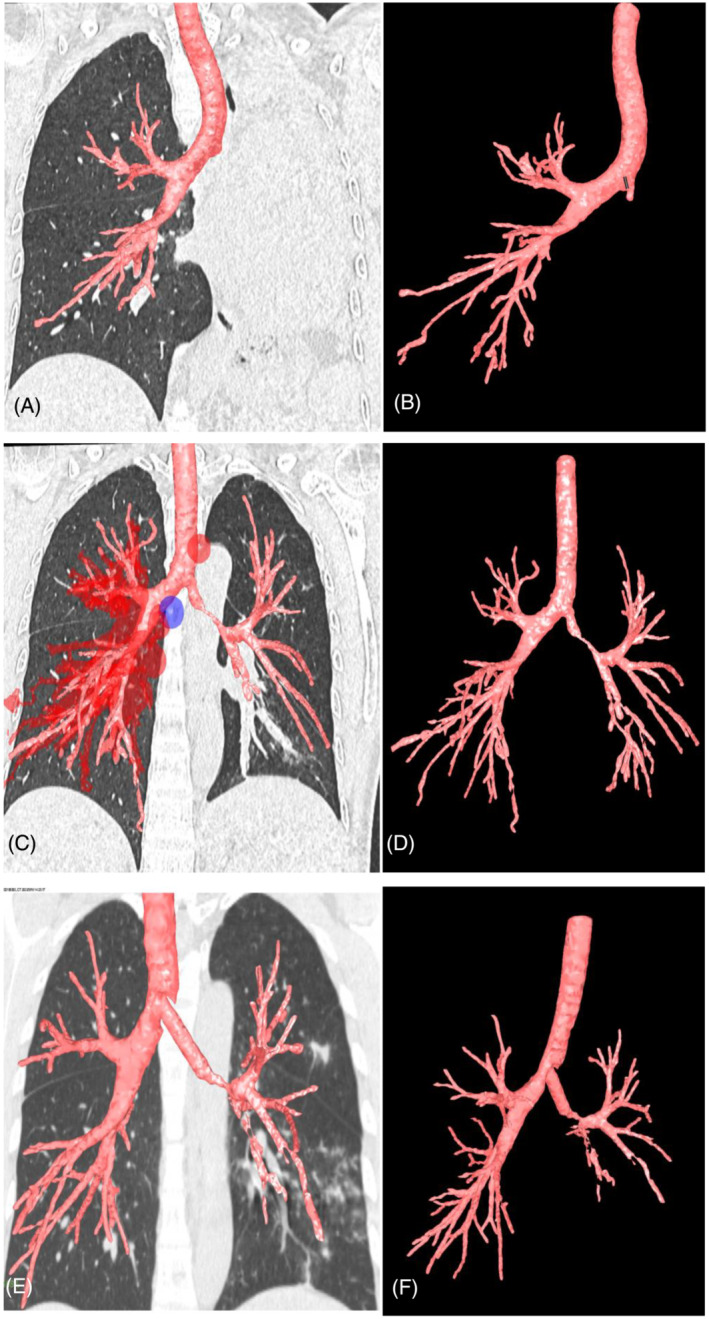
Archimedes 3D image of bronchial tree, (A, B) left main bronchial truncation, left atelectasis (Sep.23.2021); (C, D) stenosis of the left main bronchus, temporary recovery of left atelectasis, with most of the left bronchial tree visible (Oct.09.2021); (E, F) after silicone stent implantation, the left main bronchus was thicker and stiffer than before, and most of the left bronchial tree appeared (Mar.16.2022).

On September 24, 2021, YAG laser and cryotherapy were used to enlarge the occlusional left main bronchus orifice (Figure 2B). After success, balloon dilation was used to enlarge the middle and upper lumen of the left main bronchus. The middle and upper lumen of the left main bronchus was significantly enlarged (Figure 2C), and a 6 mm diameter electronic bronchoscope could be accessed. The lumen at the end of the left main bronchus was subsequently found to remain occlusional (Figure 2D). Magnetic navigation three‐dimensional imaging showed a truncated left bronchus (Figure 4A,B). The CT images and navigation images were analyzed comprehensively, and the annular ultrasound was used to explore the large vessels and determine the puncture direction, to avoid massive bleeding and perforation or injury of the heart. On October 9, 2019, the patient underwent bronchoscopic intervention for BTPNA under general anesthesia. After 10 mm of the distal left main bronchus was punctured with a FlexNeedle needle, a BRONCUS balloon dilator catheter (diameter 4 mm × 6 mm) was inserted (Figure 2D,E) and dilated with (2–8) ATM pressure once for about 2 min. There was a little bleeding. Then, puncture 10 mm forward, and repeat the above operation twice. The lumen at the end of the left main bronchus was significantly enlarged (Figure 2F). The bronchoscope (MP290F) with an external diameter of 3.0 mm can enter the left main bronchus smoothly, and the left upper and lower bronchus can be seen (Figure 3C), and the left atelectasis can be recovered (Figures 1B,C and 4C,D). On October 26, 2021, an 8 mm × 40 mm dilated balloon was used (Figure 3A,B), and a 10 mm × 40 mm covered stent was placed (Figure 3D) to slow the contraction of the tracheal lumen with expanded branches. Due to the insufficient support of the stent, the covered stent was removed and replaced with a silicone stent to maintain the open lumen of the left main bronchus (Figures 1D, 3E,F, and 4E,F). The operation was successful. After the operation, the patient's breathing was smooth and the left atelectasis recovered (see Table 1 for treatment details).

**TABLE 1:** Bronchoscopy diagnosis and treatment process and disease changes

Data	CT	Navigation imaging	Bronchial diagnosis and interventional therapy
Sep.23.2021	Left main bronchial occlusion, left atelectasis (Figure 1A)	The left main bronchus was truncated (Figure 4A,B)	The left main bronchial opening was occluded (Figure 2A)
Sep.24.2021			Laser and cryotherapy dilated the orifice and the balloon dilated the upper midsection lumen (Figure 2B,C)
Oct.09.2021	Recovery of left atelectasis (Figure 1B,C)	Stenosis of the left main bronchus (Figure 4C,D)	The vessels were explored and avoided by radial ultrasound, BTPNA was performed on the left main bronchus (Figure 2D,E)
Oct.26.2021			Covered stent placement after balloon dilation, The stent was removed due to insufficient support (Figure 3)
Nov.20.2021			Silicone stent placement was successful (Figure 3E,F)
Mar.16.2022	The Left main bronchial stent was placed (Figure 1D)	The left main bronchus was thicker than before (Figure 4E,F)	

## 4 DISCUSSION

Bronchial scar occlusion caused by bronchial tuberculosis is a serious clinical complication. The commonly used methods for bronchoscopic interventional treatment of bronchial stenosis or occlusion include laser,^14^ high‐frequency electrotomy,^15^ cryotherapy,^16^ balloon dilatation,^17^ and stent implantation.^18^ But early intervention is still needed for treatment to be effective. The success rate of common techniques in the later stage is relatively low, and the risk is very high, which is easy to induce massive bleeding and endanger the patient's life. Sometimes, surgical treatment is even needed,^19^ and atelectasis caused by bronchial occlusion has to be removed to prevent the course of the disease.

In this case, the left main bronchus was completely closed due to tuberculous injury, resulting in left atelectasis. Initially, we used a YAG laser combined with cryotherapy to open up the upper middle lumen of the left main bronchus. The bronchial walls in this area are thick and relatively safe for lasers to use. The upper middle lumen of the left main bronchus was maintained as far as possible by cryotherapy and balloon dilatation, which could ensure the extension of the therapeutic bronchoscope and pave the way for the next treatment of the lower left main bronchus. Through the working channel of the therapeutic bronchoscope, a radial ultrasound probe can be delivered to explore the surrounding large vessels, which can be avoided during forwarding puncture with a dedicated FlexNeedle needle. With a special small balloon (4 mm × 6 mm) slowly injecting pressure to gradually expand the gap formed by the needle, the bronchial wall tearing state and early bleeding tendency can be closely observed through the balloon wall, to avoid the perforation of the bronchial wall and the injury of the large blood vessels. In this process, the C‐arm + X‐ray also indirectly guides the correct puncture direction of the FlexNeedle needle, reducing the risk of the operation and improving the success rate of the operation. Based on CT scan, Archimedes magnetic navigation imaging software virtual left main bronchus images before and after patency^20^ and further evaluated the changes of left main bronchus after silicone stent implantation.

When the occlusion of the left main bronchus causes complete left pulmonary atelectasis, all the major vessels of the left lung are clustered in the hilum. CT scan shows that the occluding left main bronchus is linear and indirect in the gap between the vessels. At this point, any intervention with a bronchoscope is likely to puncture the bronchial wall and great vessels, causing massive bleeding or acute mediastinal emphysema, all of which can be fatal. Although BTPNA was successfully used in this case, the left main bronchus was opened and the patient recovered from left atelectasis, many details need to be further discussed and studied, including the popularization of the operation experience.

BTPNA technique can be used as a penetrating therapy for bronchial occlusion in clinical practice. But this technique must be performed by a trained and experienced bronchoscopist.

## CONFLICT OF INTEREST

All authors declare no conflict of interest.

**List of abbreviations**
BTPNA: Bronchoscopic TransParenchymal Nodule Access
E: ethambutol
H: isoniazid
R: rifampicin
Z: pyrazinamide

## ETHICS STATEMENT

The technology has been reviewed and approved by the hospital's medical ethics committee. The intention and purpose of BTPNA therapy were explained to the patient and family, and informed consent was signed by the patient and family.

## AUTHOR CONTRIBUTIONS

Qiusheng Jing was responsible for full text writing, all data collection, technical operation, risk assessment, Image making, and literature retrieval. Zhimin Hu was responsible for the late technical operation (BTPNA). Mingdi Wu, Min Liu, Panli Yu, and Ling Sun were responsible for technical assistance and other work. Xianxiang Chen was responsible for data review and writing guidance.

## Data Availability

Research data are not shared.
